# Outcomes of an integrated knowledge translation approach in five African countries: a mixed-methods comparative case study

**DOI:** 10.1186/s12961-024-01256-x

**Published:** 2024-12-10

**Authors:** Kerstin Sell, Eva Rehfuess, Jimmy Osuret, Esther Bayiga-Zziwa, Bezinash Geremew, Lisa Pfadenhauer

**Affiliations:** 1 Chair of Public Health and Health Services Research, IBE, Faculty of Medicine, LMU Munich, Elisabeth-Winterhalter-Weg 6, 81377 Munich, Germany; 2 Pettenkofer School of Public Health, Munich, Germany; 3 Department of Disease Control and Environmental Health, School of Public Health, College of Health Sciences, Makerere University, Kampala, Uganda; 4 Non-Communicable Diseases Directorate, Armauer Hansen Research Institute, Addis Ababa, Ethiopia; 5 Harvard T.H. Chan School of Public Health, Boston, USA

**Keywords:** Public health, Integrated knowledge translation, Evaluation, Implementation, Noncommunicable diseases, Uganda, Rwanda, Malawi, South Africa, Ethiopia

## Abstract

**Background:**

Integrated knowledge translation (IKT) aims to enhance evidence-informed decision-making in public health and healthcare by establishing continuous relationships between researchers and knowledge users, in particular decision-makers. The Collaboration for Evidence-Based Healthcare and Public Health in Africa (CEBHA+) undertook research on noncommunicable diseases in Ethiopia, Malawi, Rwanda, South Africa and Uganda. Alongside the research activities, we implemented an IKT approach, which entailed training and the development and implementation of site-specific IKT strategies. We evaluated these strategies according to a predefined programme theory.

**Methods:**

Drawing on our published protocol (https://rdcu.be/dyfBP), we interviewed and surveyed CEBHA+ researchers and their decision-making counterparts during two project stages (3/2020–2/2021; 9/2022–5/2023) and collected IKT-related documents. Transcripts and documents were analysed using qualitative content analysis and surveys were analysed descriptively, with subsequent integration, cross-case analysis and revision of the programme theory.

**Results:**

A total of 36 researchers and 19 decision-makers participated in surveys, focus groups and/or interviews, and we collected 92 documents. Relationship building, capacity building and collaborative research were the most proximal intervention outcomes: CEBHA+ researchers and their counterparts built mutual appreciation and partnerships, accessed contacts and networks, and expanded skills in conducting and using research and in IKT. The level of trust between partners varied. Intermediate outcomes were changes in attitudes and knowledge; beyond the conceptualization in our initial programme theory, researchers substantially increased their understanding of the decision-making context and developed a vision for “research impact”. While it was challenging to evaluate distal outcomes, the IKT approach was linked to the production of research perceived as addressing local priorities and being highly applicable and contextualized, and some consideration of evidence among decision-makers. Unintended effects included high opportunity costs associated with undertaking IKT. An unanticipated outcome was the heightened interest of the research funder in policy engagement. Our updated programme theory constitutes a low-level theory for IKT.

**Conclusions:**

Whilst this study faced many challenges common to the evaluation of knowledge translation interventions, it presents rich, theory-informed insights into IKT outcomes. These are based on documented IKT activities and participants’ views, particularly in-depth insights of researchers’ experiences with implementing the CEBHA+ IKT approach.

**Supplementary Information:**

The online version contains supplementary material available at 10.1186/s12961-024-01256-x.

## Background

Integrated knowledge translation (IKT) describes an ongoing relationship between researchers and knowledge users and constitutes one approach to enhancing evidence-informed decision-making (EIDM) in healthcare and public health [1, 2]. IKT goes beyond “simple” knowledge translation (KT), originally defined as the “synthesis, exchange, and application of knowledge by relevant stakeholders to accelerate the benefits of global and local innovation in strengthening health systems and improving people’s health” [3]. KT encompasses the production and utilization of knowledge in a cyclical knowledge-to-action process [4] whilst IKT underpins this and emphasizes the building of continuous relationships between researchers and knowledge users, which are considered key to fostering (i) evidence uptake, (ii) the production of relevant research and (iii) conditions that facilitate EIDM [5].

IKT is distinct from other collaborative research approaches by focusing on knowledge users with the authority to implement policy or practice changes [6]. This emphasis on involving decision-makers, including policy-makers, sets IKT apart from, for example, community-based participatory research [7] and engaged scholarship [8]. Nevertheless, all these approaches are similar in their intent to democratize knowledge creation and use, and in that they require substantial time and resources [8].

Interest in IKT has increased, including among research funders who view the involvement of knowledge users and KT activities as critical for achieving research impact [9, 10], which serves as a justification for public funding of research [11]. Whilst the promises of IKT are widely acknowledged, until recently IKT outcomes have rarely been evaluated [1]. The reasons include political and logistical challenges [12], and lack of conceptual clarity, evaluation tools and guidance [13]. Since our research was conceptualized, more frameworks for KT and IKT [14–16] and tools to evaluate research partnerships including IKT have become available [17–19] and several evidence syntheses of available (I)KT evaluations and descriptions have been undertaken [1, 20], including in African settings [2, 21–23]. Edwards et al. (2019) emphasize the potential of IKT for African health research settings; they identified the sustained engagement of knowledge users as a key facilitator to KT efforts and found that KT strategies drawing on integrated efforts or longer-term exchange featured prominently among existing strategies [23]. However, they noted that (I)KT strategies were still underdescribed, as also noted internationally [1]. They also concluded that there remains a “persistently unclear change pathway between research, KT strategies and policy formulation” [23]. This underpins that, paradoxically, EIDM lacks a rigorous evidence base for effective strategies [12].

Despite emerging evidence that IKT is associated with improved capacity to use evidence among knowledge users and production of more relevant research, empirical research on IKT outcomes is still limited [1, 5]. No study to date has examined IKT outcomes across multiple African countries, and research employing theoretical frameworks addressing this gap has been strongly recommended [23].

### Objectives

We describe proximal, intermediate and distal outcomes of the Collaboration for Evidence-Based Healthcare and Public Health in Africa (CEBHA+) IKT approach. As secondary objectives, we explore variation across the five African sites and revisit the CEBHA+ IKT programme theory.

## Methods

### Research approach and paradigm

We undertook a mixed methods comparative case study employing semi-structured interviews and an online survey at two evaluation stages (subsequently “waves”: 3/2020–2/2021 for the first wave and 9/2022–5/2023 for the second wave). Documents were collected until June 2023. Our methods largely followed our protocol [24], but we had to make some adaptations linked to the coronavirus disease 2019 (COVID-19) pandemic and staff turnover. These included a change from face-to-face to online interviews during the first wave; during the second wave, they entailed a switch from interviews to online focus group discussions (FGDs) and foregoing interviews with decision-makers.

Our work was informed by a critical realist research paradigm. This is rooted in an understanding an objective reality exists and that this can be studied using scientific methods – whilst such knowledge is subjective and socially constructed reflecting “epistemological relativism and ontological realism” [25]. Evaluations rooted in critical realism are intended to generate understanding about causal mechanisms and context and incorporate programme theories [26] to delineate “what works, for whom, why and in what circumstances” [27].

### Intervention description and implementation

This study was undertaken in the context of the Collaboration for Evidence-Based Healthcare and Public Health in Africa (CEBHA+). The consortium comprised nine academic institutions in Ethiopia, Germany, Malawi, Rwanda, South Africa and Uganda, and was funded by the German Federal Ministry of Education and Research from 2017 to 2023. It aimed to conduct policy-relevant and practice-relevant research on the prevention and integrated care of noncommunicable diseases (NCDs), notably diabetes and hypertension, and road traffic injuries. The German project partner LMU Munich was tasked with leading on the development and evaluation of the CEBHA+ IKT approach.

The CEBHA+ IKT approach entailed training and guidance on IKT and the implementation of IKT strategies at the five African sites. A detailed description [28] and reflections about the IKT experiences in CEBHA+ were previously published [29–32]. Systematic approaches for mapping and analysing stakeholders^1^ and for developing and implementing an “IKT strategy” were key tools [33, 34]. Both tools enabled researchers to strategically collaborate with partners from policy and practice from the start of the project and tailor IKT activities to their needs and preferences, that is by choosing the appropriate timing, messenger, message, medium and format for engagement. Initially, the tools for planning IKT activities were utilized widely but less so at later stages in the project [28]. Ongoing IKT activities were coordinated by one or two “IKT focal points” per CEBHA+ institution. IKT focal points and IKT evaluators (the “IKT team”) met quarterly online to discuss IKT experiences, forming a community of practice.

### Programme theory

**Fig. 1:**
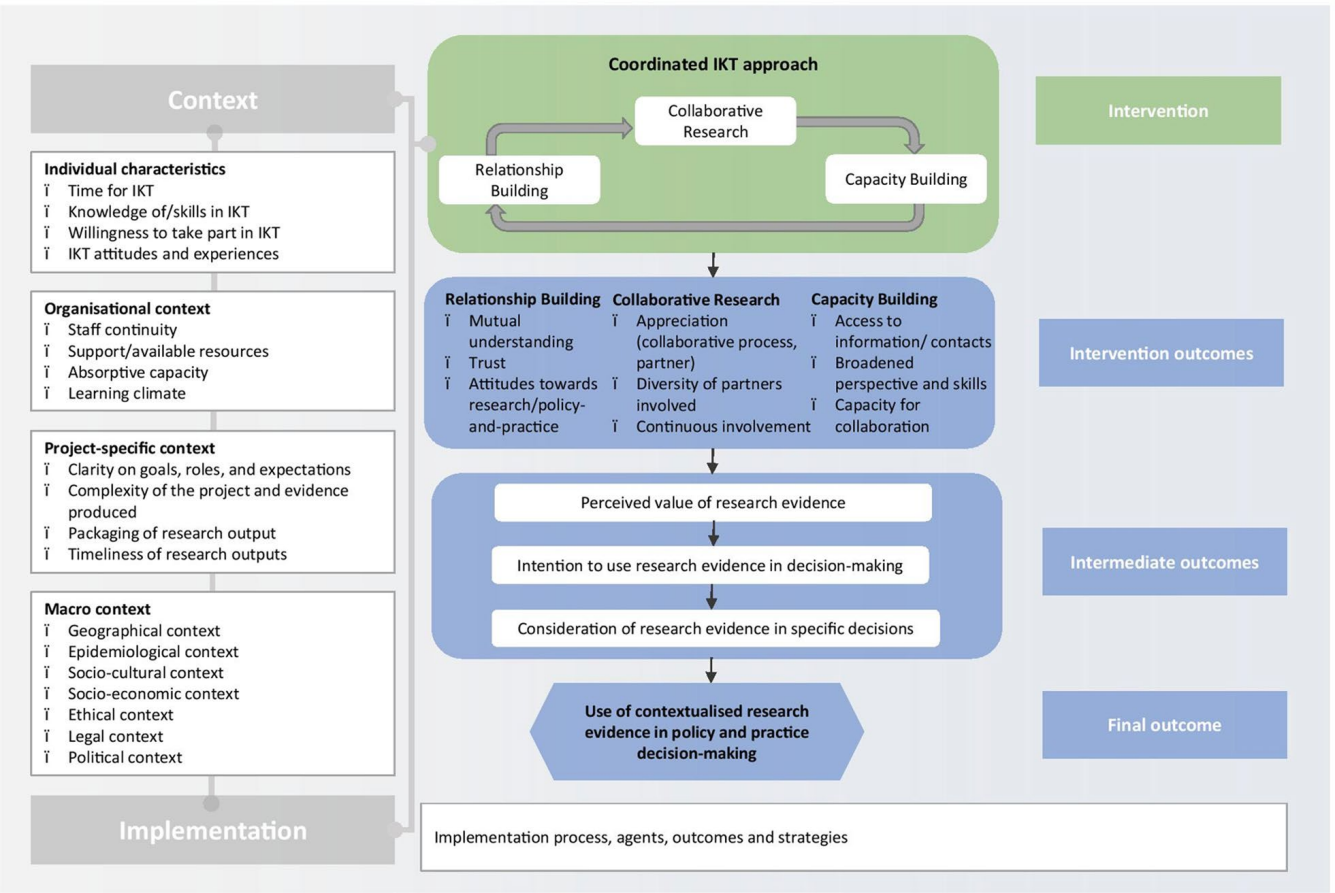
Initial programme theory of the CEBHA+ IKT approach

We were not able to identify an existing programme theory for IKT that fit our scope. Programme theories guide evaluation by making assumptions about underlying mechanisms explicit [35]. We therefore developed a programme theory, informed by a scoping review of IKT evaluations [1] and further purposively selected literature: hypothesized proximal outcomes were relationship building, capacity building and collaborative research, as suggested by realist IKT theory [36]. Intermediate outcomes included changes in attitudes and evidence use behaviour of decision-makers [5, 36]. Ultimately, we hypothesized that IKT would lead to increased use of contextualized research evidence in decision-making (conceptualized as the final outcome) (Fig. 1).

### Intervention context

KT structures and culture pre-existed at all sites but varied considerably [28]. This included a strong focus on EIDM among academic institutions in South Africa, government policy mandating community engagement in research in Rwanda, a KT platform in Malawi [37] and long-time practice of engaging stakeholders in Uganda. The research environment was shaped by resource constraints, with research depending on foreign funders whose research agenda is often misaligned with local priorities [22].

### Sampling strategy

All CEBHA+ researchers involved in IKT were eligible for inclusion. Policy and practice partners were eligible when they collaborated actively, were over 18 years old and English speaking. We did not undertake a sample size calculation, nor did we define a cut-off for inclusion of participants when data saturation would be reached due to the generally limited number of eligible individuals.

### Data collection methods

Data collection for the first wave depended on the timing of the respective approval from local institutional review boards, resulting in a long data collection phase (3/2020–2/2021). The first wave therefore does not constitute a baseline assessment. Interviews were conducted in-person in Malawi. Following the onset of the COVID-19 pandemic, interviews were almost exclusively conducted online, except for interviews with Ugandan stakeholders, some of which were conducted in person by trained local staff. Researchers were contacted via email and invited to the interview and survey. Stakeholders were invited by their respective CEBHA+ contact who forwarded a formal invitation letter. Data collection for the second wave took place during project finalization (9/2022–5/2023).

### Data collection instruments

#### Instrument development

As we were not able to identify suitable instruments, we developed CEBHA+ surveys and interview guides based on relevant survey constructs from existing instruments, identified in a purposive search and drawing on our programme theory, as reported elsewhere [24]. Surveys consisted of a demographic survey, followed by 69 questions for stakeholders and 62 for researchers in 8 dimensions, corresponding with the programme theory constructs. Semi-structured interview guides consisted of nine topic areas, each including multiple probing questions on the constructs. We developed and pilot-tested stakeholder and researcher versions. All instruments are available elsewhere [24].

### Documents

We included internal and external IKT-related documents. IKT updates, the main source of internal documents, were submitted by the IKT implementers every 3 months and replaced monitoring with a more extensive bespoke tool, which proved infeasible as CEBHA+ researchers perceived the workload for documentation as too overwhelming [28]. The IKT updates included an overview of IKT activities in the reporting period and insights or stakeholder feedback obtained but no formal monitoring (for example the number of outputs distributed or downloaded, number of meeting attendants). Some monitoring data was available from meeting minutes. External documents mentioned by CEBHA+ researchers (for example guidelines, policy documents) were included and supplemented with documents identified in a purposive google search.

### Data processing

Interviews were recorded using the online conference tools’ recording function or a handheld recorder (OLYMPUS LS-P1). The interviewer wrote a memo after every interview. Recordings were securely stored on an encrypted device and transcribed verbatim by Audiotranskription, a transcription service complying with required levels of data protection [38]. Transcripts were provided to the interviewees for review. Original audio files were deleted after transcription. Transcripts and documents were pseudonymized and analyzed in ATLAS.ti [39]. We conducted surveys in the online tool Limesurvey [40]. Anonymous survey data were exported and analysed in Microsoft Excel.

### Data analysis

We considered the five African sites as “cases”, analysing data separately per case (where possible) and subsequently comparing results across cases [41]. We initially analysed data from surveys, interviews, documents and focus groups separately, integrating these over time [42].

Quantitative survey data were analysed descriptively, by subgroup (researchers, stakeholders). For qualitative content analysis, domains and constructs of the programme theory served as the initial coding frame [24, 43]. One researcher (KS) coded interviews and documents, applying codes deductively and inductively developing new codes and categories. Coauthors (JO, EB, LMP) reviewed the codebook and applied the coding frame to one interview each, which led to further refinement. One researcher (KS) applied the final coding frame to the entirety of included material and double-coded the material. A subset of the coding was reviewed by a second researcher.

One researcher (KS) then reviewed categories, associated codes and quantitative data by site and across sites and drafted the initial results section through “theoretical integration” [42] and “abstraction and retroduction” [25], which was reviewed by coauthors, discussed and iteratively revised. Throughout this process, we adapted and expanded programme theory subconstructs.

Scientific rigour was enhanced by following the Standards for Reporting Qualitative Research [44] and using the ASSESS reporting tool, intended to harmonize reporting of implementation-focused studies employing mixed methodologies [45] (Supplement 1).

### Researcher characteristics and reflexivity

KS, ER and LMP are based at a well-resourced university in Germany. They are from non-migrant, academic backgrounds and have some to moderate research experience in African settings. JO, EB and BG are from Uganda and Ethiopia, respectively, with substantial research experience in their countries and some research experience in Europe (JO) and the United States (BG). Whilst we aimed to address the issues around power, “foreign gaze and pose” [46] in this collaborative work, we were unable to achieve this to the extent we would have aspired to as the write-up of this publication occurred after project funding had expired. Thus, our results are likely to reflect the social positions and experience of the German authors more strongly than those of the other authors.

Three authors were part of a small group of “IKT experts” that developed and launched the IKT approach (LMP, ER) and provided continuous support for IKT implementers (KS). Whilst this level of involvement in IKT implementation was critical for enabling a deeper understanding of the implementation process, it blurs the lines between evaluators and implementers.

### Ethics approval

This study was approved by the Ethics Committee of Armauer Hansen Research Institute (AHRI), Ethiopia (PO/31/20), the Ethics Review Committee of the Ludwig-Maximilians Universität München, Germany (19–633), the Research and Ethics Committee at the Kamuzu University of Health Sciences, Malawi (P.11/19/2850), the Rwanda National Ethics Committee (074/RNEC/2020), the Human Research Ethics Committee of the University of Cape Town, South Africa (026/2020) and the Ethics Committee of Makerere University, Uganda (Protocol 469). Informed voluntary consent was sought both in writing and verbally from all participants.

## Results

### Characteristics of study participants

A total of 36 researchers and 19 decision-makers participated in one or both waves (Table 1). In the first wave, 25 researchers and 10 decision-makers participated. In this wave, no decision-makers from Rwanda and Ethiopia were involved as we deemed it unethical to approach them at the height of the COVID-19 pandemic. In 2022, no decision-makers from South Africa were approached for logistical reasons. In the second wave, 27 researchers were surveyed, of which seven participated in a focus group discussion.

**Table 1:** Study participants

	CEBHA + researchers		Decision-makers	
	First wave	Second wave	First wave	Second wave
Total *N* participants	25	27	10	11
Survey *n* responses^a^	24	27	7	11
Survey response rate	24/26 (92%)	27/32 (84%)	7/14 (50%)	11/16 (69%)
Interview	25	–	8	–
FGD	–	8	–	–
Both (survey and interview or FGD)	23	7	5	–
Country				
Ethiopia	2	5	0	2
Malawi	5	6	4	3
Rwanda	6	6	0	4
South Africa	7	7	1	0
Uganda	4	3	5	2
Gender^3^				
Female	16	13	1	4
Male	7	13	6	7
Prefer not to say	1	0	0	0
Highest educational background _b_				
Doctorate	11	10	1	1
Master’s degree	10	12	4	9
Professional degree	3	4	1	1
Bachelor’s degree	0	1	1	0
Professional background _b_				
Public health	11	13	1	6
Medicine	10	7	3	1
Epidemiology	2	0	0	0
Other	1	5	3	4
Institution _b_				
University	19	18	0	0
Research institute (not within a university)	5	7	0	1
Government department	0	1	5	8
Regional or local health authority	0	0	1	0
Nongovernmental organization	0	0	0	2
Time at this institution _b_				
One year or less	1	0	0	0
2 or 3 years	8	4	1	0
4 or 5 years	3	7	1	3
6–10 years	6	6	1	5
11–15 years	1	7	1	2
More than 15 years	4	3	3	1
Overall work experience in the field _b_				
5 years or less	2	5	1	1
6–10 years	9	3	2	4
11–15 years	7	8	1	4
More than 15 years	5	11	3	2

*FGD* focus group discussion; ^a^participant numbers include incomplete forms (*n* = 1; except in the DM 2020 wave); ^b^based on sociodemographic information in survey responses

### Included documents

We included 92 IKT-related internal and external documents (Table 2).

**Table 2:** Included documents

	Ethiopia	Malawi	Rwanda	South Africa	Uganda	Other	Total
IKT strategies (including revised versions)	1	3	1	3	2		10
IKT “updates”	9	11	9	7	8		44
Documentation of IKT activities (for example meeting minutes)	1	5	0	1	7		14
IKT team call minutes						17	17
External documents (for example National Strategic Plans for NCDs or Road Safety, funder-initiated evaluation, WHO guideline, transport policy)			1	2	2	2	7
All documents							92

### Proximal outcomes

We identified proximal outcomes related to capacity building, relationship building and collaborative research.

### Capacity building

Capacity building is a multilevel concept [47]. In our initial programme theory, hypothesized outcomes included only individual-level outcomes, notably “access to information and contacts”, “broadened perspective and skills”, and “capacity for collaboration”. Below, we expand these to capacity building at the (i) individual and (ii) organizational level as well as capacity building contributing to an (iii) enabling environment [47].

### Individual-level capacity building

#### Research and research use skills

**Fig. 2:**
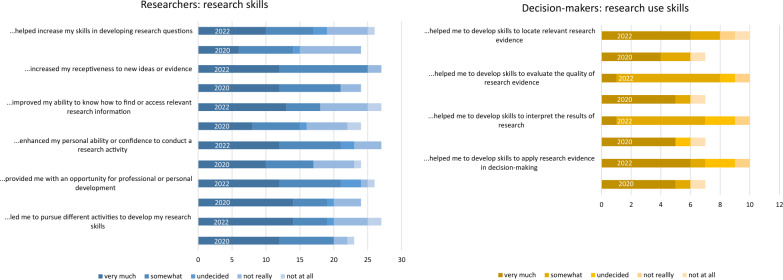
Research skills and research use skills, in 2020 and 2022, respectively

Researchers reported mostly positive results with respect to research skills gained (Fig. 2). Approximately 80% of researchers indicated that the collaboration had increased their receptiveness to new ideas or evidence [(*n* = 21 (2020), *n* = 25 (2022)], and provided them with an opportunity for personal or professional development [*n* = 19 (2020), *n* = 21 (2022)]. Fewer respondents indicated that they had improved their capacity to develop research questions, conduct research and improve their ability to access relevant research information. In qualitative data, there was little reference to research skills gained.

The majority of decision-makers indicated that the collaboration had helped them increase their research use skills, that is to locate, evaluate, interpret and apply research evidence (Fig. 2). In qualitative data, there was no reference to evidence use skills, except for researchers in Uganda and Rwanda reporting that decision-makers were interested in learning research methods.

### IKT skills

IKT skills relate to the identification of stakeholders, development and implementation of an IKT strategy and general stakeholder engagement (Table 3). Most researchers already had substantial experience in stakeholder engagement as part of their work routine. They reported high levels of comfort and skills to engage in the research collaboration but few had received prior KT training. At the second wave, 88% of surveyed researchers indicated that the IKT approach helped them to make deliberate decisions about stakeholder engagement, 92% felt comfortable advising colleagues on this, and 77% would recommend an IKT approach in future projects.

**Table 3:** IKT skills

IKT skills subcategory	Skills	Detail and exemplifying quotes
Stakeholder identification skills	Skills to identify, map, prioritize and analyse stakeholders	“Yes, so, when we did the stakeholder mapping, we specifically said what we needed each stakeholder for because they have different strengths and […] areas of expertise.” (MW RS 4) “But also, which stakeholders can also negatively influence how your research/ […] how well it's received.” (MW RS 2)
IKT strategy development and implementation skills	Skills to choose the appropriate timing for IKT	Includes consideration of current stakeholder priorities and timeliness of responses: “You can’t ignore […] what issues are priorities at that time. So, like a month before the election, people are very, very busy in every Ministry […]/ So, you can’t go there and then say, ‘I want to talk about salt reduction, and, you know.’ You have to sense the time.” (MW RS 4) “[…] within CEBHA and also within other projects, the one thing that I’ve learned is around timeliness of responses and engagements and knowing when is the right time to contact and ask for information and also the right time for putting things forward […]. And so I think key to keeping that relationship going is timeliness of information […]/ people at the policy level are inundated with a lot of requests and lots of different researchers coming to them from all different fronts. So you must find ways of not also adding to that burden.” (SA RS 7)
		Includes consideration of when not to engage stakeholders: “And there’s been moments where we’ve had to make decisions that actually maybe now is not a good time to actually engage that stakeholder about this particular issue because, in a month’s time, we want to engage them about something else.” (SA RS 4)
	Skills to choose the appropriate messenger for IKT	Included various strategies: – identifying a team member who was “exceptionally good at forming personal relationships” (Ethiopia) – assigning messengers based on geographical proximity (Malawi) – using researchers’ long-standing contacts (all teams) – enlisting stakeholders as messengers, for example lower rank staff within the same organization to follow-up with senior staff (Uganda) – accessing new contacts through existing stakeholder contacts (Rwanda, South Africa, Malawi) “[… our messenger] varies from our communication to another one. […] For example, [… researcher] who has been working with a specific stakeholder like RBC, the Department of NCDs, [it is therefore] easier to let him continue in that way and keep enforcing the ties that are existing between the two institutions.” (RW RS 2)
	Skills to choose the appropriate forum and medium for IKT	Included skills in developing issue briefs Included gauging whether stakeholders preferred further in-person meetings after initial face-to-face meetings and using conferences and trainings strategically as for a for engagement
General stakeholder engagement skills	Understanding stakeholder needs and interests	Closely linked to skills in stakeholder mapping and IKT strategy development: Understanding of stakeholders’ motivation helped researchers to choose an appropriate engagement strategy. Strategies included: – highlighting of the “mutual benefit” of the collaboration – showing appreciation for stakeholders’ input, for example by involving them as guest speakers – monetary or in-kind incentives: “So for a lot of governmental departments, they will meet you if there’s an allowance involved. So if you have a Skype meeting, for example, they don't really benefit anything. We can't get their transport reimbursed.” (MW RS 2)
	Dealing with staff turnover	Ugandan and South African teams strategically established multiple connections to address this challenge: “[…] if we are contacting Ministry of Health, […] we should not focus on one person. […] This has been a lesson to us. Never have just one person involved as a stakeholder from one organization.” (UG RS 1) “And to have multiple members of our staff know about those and be introduced to those relationships, so that we never are in a situation where we have a fractured relationship, […] that’s been really important in terms of our strategy, irrespective of who’s maintaining the relationships that were not overly reliant on individuals.” (SA RS 3)
	Dealing with stakeholder power	This was actively considered at the stakeholder mapping phase but was otherwise rarely addressed explicitly. Skills in navigating power imbalances in the research collaboration are still evident implicitly through the variety of strategies employed by researchers to deal with this challenge: – making concessions to high-level decision-makers to accommodate their schedule – considering hierarchies to choose an appropriate messenger – balancing of researchers’ support of the decision-maker’s agenda – managing of expectations regarding such tasks and available resources: “[Decision-makers] will be very excited about the project. But then they will have ideas on what it is they want to add. And you’re like, ‘No. […] it would be wonderful to do that. But that’s not part of my plan. And I actually don't have the resources for that.’ So it’s something that one has to do. I think be wise. How do you say it without saying [that you are] only interested in your own activity, which you can fund.” (UG RS 2)

IKT skills were not investigated in our survey, but featured strongly in qualitative data. In general, researchers from all countries had developed substantial skills in developing and implementing an IKT strategy and honed general stakeholder engagement skills (Table 3). However, skills related to the timing of engagements and deprioritization of stakeholders were only mentioned as important by three interviewees from South Africa. Knowledge of IKT as a theoretical concept and an area of research remained more ambiguous with researchers frequently describing that they were on a “learning curve”.

### Organizational-level capacity building

Organizational capacity building occurred to a variable extent. IKT staff time was underbudgeted for in four countries, which meant that IKT focal points undertook IKT in addition to other work (Ethiopia, Rwanda, Uganda). In Malawi, an intern coordinated IKT activities. The resulting limited staff time for IKT, in combination with insufficient IKT training and staff turnover, presented challenges for organizational capacity building.

Nevertheless, efforts to build structures for organizational capacity for EIDM included the development of an evidence-based public health course which was run in all CEBHA+ countries [48]. In South Africa, this complemented one institution’s existing KT and issue brief courses [33, 34, 49, 50], which were attended by some CEBHA+ researchers. The Ethiopian team’s collaboration with the new knowledge management unit at AHRI and the KT unit at the Ethiopian Public Health Institute contributed to cross-institutional capacity building.

### Capacity building for an enabling environment

Some IKT efforts contributed to an enabling environment for KT beyond the participating organizations. The Malawi team partially revived an existing KT platform. In South Africa, the NCD symposium (2020) presented an opportunity for a variety of actors to network and access local NCD research. The Ethiopian team initiated a longitudinal training and mentorship for junior researchers who undertook systematic reviews on topics prioritized by the Ministry of Health (MOH). Of note, none of these efforts were CEBHA+ deliverables. Instead, capacity building for an enabling environment addressed local needs (that is for training or networking opportunities).

### Relationship building

Relationship-building is a process of establishing, strengthening and maintaining relationships. In our initial programme theory, outcomes linked to relationship building were mutual understanding, trust and attitudes. These were revised to include (i) appreciation, (ii) trust, (iii) partnership formation and (iv) access to contacts and networks.

### Appreciation

In quantitative and qualitative data, appreciation for the collaboration, the respective partner and for early engagement featured prominently. Most survey respondents agreed that contributions of partners were valued, that the collaboration incorporated broad perspectives and added value to the research. Agreement was lower for statements about acknowledgement in project reports and of partners’ views (Table 4).

**Table 4:** Relationship-building outcomes: appreciation, trust

	Researchers		Decision-makers	
	2020	2022	2020	2022
	*N* (%) Agree	*N* (%) Agree	*N*/total agree	*N*/total agree
Appreciation				
The individuals involved represent a broad range of perspectives^a^	21 (91)	24 (89)	6/7	7/8
CEBHA+ partners value my contributions^a^	19 (83)	22 (81)	6/7	7/8
All partners are acknowledged in CEBHA + project documents (for example reports, publications)^a^	15 (65)	20 (74)	4/7	6/8
The collaboration between stakeholders and researchers in CEBHA+ added value to the ongoing research^a^	21 (91)	26 (96)	6/7	7/8
…I feel that my views are heard^b^	13 (54)	13 (48)	7/7	6/10
Trust				
…the collaborating partners trust each other^b^	13 (54)	13 (48)	3/7	7/10
…I am able to express my views freely^b^	20 (83)	19 (70)	6/7	6/10

Question format: ^a^yes/no/do not know, ^b^check all that apply

Decision-makers strongly valued being involved by researchers early on in the research process and were content to provide input, which facilitated later work:

“[Partners were] part of our planning meetings. They were the ones who told us you haven’t thought about this, you haven’t thought about that, you haven’t included this person. So, it was very easy to go back to them once we started collecting data, and say, ‘[…] we're having a problem in this area. Can you help?’ They were very keen to help because they already knew about us.” (MW RS 4)

Stakeholders appreciated the collaborative approach as it contrasted with usual practice:

“We hear from [community stakeholders] that the CEBHA project is a good research because it has involved them, it is not the same as other research projects which come and collect data without involving them. They said if other studies are conducted like CEBHA it would be fine. So for them they are appreciating it.” (RW RS 5)

In Uganda, stakeholders appreciated the CEBHA+ meetings for providing a forum for conversation between various road transport stakeholders who would not normally talk to each.

### Trust

Survey participants mostly agreed that they were able to express their views freely in the collaboration but substantially fewer participants thought that collaborating partners trusted each other (Table 4).

Qualitative data provided a more nuanced picture. Without prompting, decision-makers often referred to trust, along with professionalism and respect, as a key relationship characteristic:

“I think it’s a trusted relationship. It’s a trusted one. It’s both professional and friendship.” (MW SH 4)

A substantial level of trust is evident from one Ugandan decision-maker’s statement:

“[I]n most cases I tell [Researcher] what I think, irrespective of what the government position might be. Or the governmental shortcomings might be. So of course I tell her the challenges we are having, and some of them we discuss. Political relationship of this country. […] how road safety doesn’t get an investment.” (UG SH 2)

Instances of decision-maker initiated engagement, for example to discuss evidence gaps during the COVID-19 pandemic, reflect that CEBHA+ researchers were seen as trusted sources of information.

In Uganda and South Africa, decision-makers built trust with members of the broader research team over time, beyond the initial senior researcher contact. However, despite individual examples of trust between partners, other relationships were characterized by low levels of trust: survey questions about honest communication about needs, constraints or organizational realities received low endorsement rates. Fear of a “hidden agenda” was frequently referred to by interviewees. Ugandan decision-makers mentioned “fear of losing face” as a reason for why partners may not trust each other enough to communicate transparently.

### Partnership formation

CEBHA+ teams formed partnerships with varying levels of partnership strength.

The understanding of “partnership” was ambiguous. Ugandan decision-makers appeared to understand “partnership” mainly as a source of financial support. This may explain why, in 2022, three decision-makers (from Ethiopia and Rwanda) reported that they did not think they had established a partnership with CEBHA+ researchers.

Others used the term “partnership” or referred to partners as a “team”, being “part of the project” or “on board”. Some particularly strong partnerships were rooted in longstanding connections:

“I’m always available for [Researcher] to call me. I’m always available when [Researcher] calls the workshop. And I don’t think I’ve ever, ever not responded to [their] call.” (UG SH 2)

Interviewees in South Africa described one decision-maker as their “champion”, a term introduced in IKT training:

“[…] I would say the person who had made CEBHA more […] acceptable and implementable in South Africa, yeah, she was the champion. […] unfortunately, because of COVID, she was also very, very busy, and she really gave more than what a stakeholder of her calibre would, but it’s because she had an interest of the kind of work that we’re doing, the knowledge, the science.” (SA RS 2)

In IKT documents, partnership formation was evident from the continuous presence of certain decision-makers in project meetings.

### Access to contacts and networks

Researchers and decision-makers gained access to a wide range of new contacts and networks. A majority of survey participants agreed that the collaboration had “very much” or “somewhat” improved their access to contacts.

Researchers gained access to EIDM-focused networks, such as “Evident” and the “African Institute for Development Policy” in Malawi, and to some high-level decision-makers:

“And then, we actually […] had access to the Minister of Health who was also at the [South African NCD] symposium and gave a talk […] which is a very (laughing) rare thing to happen.” (SA RS 1)

Researchers and their institutions were thought to benefit from these networks in the future:

“Actually, CEBHA could initiate the network, but […] AHRI will be in position to, you know, continue the networking or the relationship with different stakeholders. […] In IKT, you will build a network. It’s not only for the CEBHA, but even for my personal career, networking has some impacts.” (ET RS 1)

### Collaborative research

In collaborative research, researchers and stakeholders work together over all or multiple phases of a research project, ideally in a continuous manner. In our initial programme theory, outcomes linked to collaborative research were appreciation, diversity of partners, and continuous involvement, which were reduced to just one subcategory.

### Continuous involvement

Researchers interacted with stakeholders most frequently in early and late research phases [28], except in Uganda and Rwanda, where collaboration was more continuous as it included data collection (Supplement 2).

Collaboration commenced during the priority-setting process for the project which involved decision-makers [51] and linked to the existing KT routines of CEBHA+ teams. Some early interactions with decision-makers were mandatory, for example requesting research approval. At all sites except Uganda, stakeholder engagement was part of a research method, that is for citizen science [52] and for the situational analysis [53, 54].

“[The] situation-based analysis for population-based interventions targeting risk factors of diabetes and hypertension […] required from the onset […] engaging with the stakeholders as we developed the whole data collection tools and […] process.” (SA RS 2)

Some data collection activities required facilitation by decision-makers, for example for accessing health and police records (Uganda) or insurance data (Rwanda). Some collaborative research originated from the strategic establishment of boundary spanners enabling repeated interaction: decision-makers had been asked to join the project advisory board (Uganda, South Africa) or undertook research on CEBHA+ PhD studentships (Malawi, Rwanda).

### Intermediate outcomes

Intermediate outcomes emerge from the interplay of the proximal outcomes. In our initial programme theory, we had only conceptualized decision-makers’ attitudes as intermediate outcomes. We revised this as changes in knowledge and attitudes among both decision-makers and researchers.

### Changes in knowledge

**Fig. 3:**
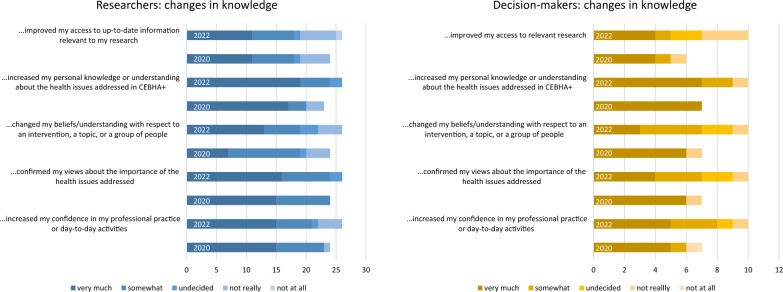
Researchers’ and decision-makers’ changes in knowledge, in 2020 and 2022, respectively

This included decision-makers learning about (CEBHA+) research and researchers learning about the decision-making context. In the survey, a majority of researchers and decision-makers indicated that the collaboration had increased their knowledge, understanding or views about the health issues addressed in CEBHA+ and enhanced their confidence in day-to-day activities (Fig. 3).

Fewer survey participants thought that the collaboration had improved their access to relevant information.

Contrastingly, interview data indicate that researchers actively sought and accessed information. This strongly improved their understanding of the decision-making context, for example regarding political mandates, priorities and post-election restructuring, and subsequently informed research activities:

“What I understand from the ministry is […] they have deliverables that they have to put through, but they don’t have the resources to do so. So [when] we kind of give them the platform […] or rather ask them what they need from us, it kind of opens up […] It makes it easier for us to get our work done when we also put forward their agenda.” (MW RS 2)

Seeking to understand decision-making priorities, the Ethiopian team conducted a needs assessment among MOH staff.

Decision-makers’ knowledge of relevant research features less prominently in interviews. In one case, they requested results from a systematic review but did not learn much as the review was “empty”. Still, seven out of eight decision-makers felt that they had become more knowledgeable about the scope of work at the research institution (2022 survey). The IKT approach further enhanced the visibility of the research institutions.

Across all groups and sites, gaining a new perspective was emphasized as a key outcome:

“[…] my interaction with the researchers […] it really opened up my mind. It pushed me to think more, to perceive things differently.” (UG SH 3)

### Changes in attitudes

We identified subtle changes in attitudes towards research evidence (decision-makers) and more pronounced attitudinal changes regarding research impact (researchers) and research collaboration (both groups). These were typically linked to existing, predominantly positive attitudes.

### Decision-maker attitudes

Decision-makers who were aware of CEBHA+ evidence generally valued this evidence and indicated that they were intending to use it (Table 5).

**Table 5:** Decision-makers’ attitudes towards research evidence

		2020	2022
	The research evidence produced in CEBHA+ …	Agree *N*/total	Agree *N*/total
Value	… is relevant to the health issues addressed by the partnership	7/7	6/6
	… is directly applicable	5/7	5/6
	… is trustworthy	7/7	6/6
	… more likely to be used in decision-making	6/7	6/6
	… more likely to have a lasting impact on public health	6/7	6/6
Intention	I intend to use CEBHA+ research evidence in my work	6/7	9/10
Consideration	Being engaged with CEBHA+ increased my active consideration of research evidence in my work	7/7	7/10

In qualitative data, decision-makers’ interest in research was apparent but this largely seemed to pre-date the CEBHA+ project.

“[…] through some of the stakeholder's meetings, we [heard that], they’re eager to get the […] study results, […] and then to see what they can improve because in the Rwandan context, […] from the administration and governance level, they are looking for anything that can [improve] what they are doing.” (RW RS 1)

It was also evident that attitudes towards research collaboration had improved among some stakeholders, often due to persistent efforts by researchers:

“[Eventually, the road traffic insurance companies] were very happy to help us, […] even someone […] who didn’t want to talk to you, they are very excited to give us information and to help us […] because it’s in their big interest […] And that’s like the approach we used to get them on board. They were like, ‘So at the end of the day, […] we are the ones who are going to gain from this project.’” (RW RS 6)

This was echoed in survey data: All decision-makers surveyed in 2022 indicated that they would reach out to the CEBHA+ researchers for future queries.

### Researcher attitudes

Many researchers embraced the “IKT idea” as a vision for research impact and became more interested in collaborating with decision-makers. Research impact was seen in both visionary and practical terms:

“It makes you […] remember not to do things in a bubble. Always consider what your long-term objectives are. So don’t do research for the sake of doing research, but always to think long term and to think who else needs to know about this. What input can they give?” (MW RS 2)

Among some researchers, this attitude was coupled with great openness to taking on decision-maker insights, for example when co-developing a logic model for integrated models of care to ensure it was relevant to both the South African and global context. Two senior researchers were described as only becoming interested in (I)KT and research collaboration through CEBHA+ and one Ethiopian researcher reported change at the level of the institution, where the benefits of systematic stakeholder engagement became recognized:

“So, now the other projects or other directorates under AHRI, they are consulting us. Or they are using us as a, you know, a link to the different stakeholders.” (ET RS 1)

The interest in IKT is also reflected in researchers adopting IKT in other projects, such as proposals and teaching (Germany, South Africa).

### Unintended and unanticipated effects

The opportunity cost of doing IKT was an unintended – yet foreseeable – outcome discussed by some researchers, leading to potentially overburdening decision-makers in resource-constrained settings:

“[I]n some ways, CEBHA has been trying to reinvent some wheels […] there’s already a lot going on in [our country] that could’ve been built on […] because the policy-makers that people are trying to engage with are all busy people. […] if the people keep saying, ‘[…] we’re going to do policy engagement. Come to our stakeholder meeting now […].’ I think that has real opportunity costs in terms of trying to take people’s time up that way.” (RS^2^)

Within the broader EIDM system, the IKT approach sparked the research funder’s interest in research impact, which was not anticipated: The CEBHA + IKT approach was mentioned multiple time as a good practice example in the funder-initiated midline evaluation and “policy engagement” became mandatory in subsequent funding lines.

### Distal outcomes

Increased use of contextualized research evidence in decision-making was initially conceptualized as the final outcome linked to the IKT approach. We rephrased this category, added the researcher dimension, the production of relevant research evidence and adapted the decision-maker dimension to consideration of contextualized research evidence, as evidence use proved conceptually challenging to evaluate.

### Production of relevant research evidence

Due to the small number of study participants, our insights into decision-makers’ views on CEBHA+ outputs remained limited. In the survey, the majority of decision-makers agreed that CEBHA+ research evidence was presented to them in an accessible format, an understandable language and a timely manner.

Two CEBHA+ projects were highlighted by decision-makers as important solutions to local priorities, for example:

“So the great initiative for diabetes that was done by [Researcher], that was important because we are not counselling our diabetic clients as we are counselling our HIV positive clients. [..] But both of them have significant morbidity and mortality. So he came with a group education initiative for diabetic patients. So that’s something that we needed.” (SA SH 5)

This study subsequently informed the local department of health and guidelines in Ethiopia, Malawi and Rwanda. Ugandan researchers and their partners identified the lack of digitized, valid road traffic crash data as a major gap. This led to a joint pilot project digitizing crash data from police and healthcare archives, which had not been planned in the original proposal. One decision-maker explained that CEBHA+ research was highly applicable but recommendations were not implemented:

“I have seen the benefits [and] the value that they could bring to the sector, to the prevention of road crashes. But yeah, I haven’t had the opportunity to implement them. […] so the applicability of research findings is they can really be done.” (UG SH 2)

Additionally, beyond what was planned in the original proposal, CEBHA+ funding enabled conducting ten rapid reviews on stakeholder-prioritized COVID-19 topics in South Africa and five systematic reviews on various MOH-prioritized questions in Ethiopia.

Other qualitative data pointed to more ambiguity regarding the relevance of research evidence produced in CEBHA+. In Rwanda, the CEBHA + team used old NCD data due to the tight project timeline despite the MOH requesting use of newer data. Similarly, a South African researcher was only able to interview very few decision-makers for their situational analysis, limiting its relevance. In Malawi and Ethiopia, the CEBHA+ priorities defined with some decision-maker involvement in 2013 [51] no longer matched the current research priorities:

“We had already met with the Ministry of Health and told them about our community level screening […]. And they didn’t say a thing. And then when we found out […] that they had been doing this already for 3 years in Addis Ababa. So I mean our project […] was, well, yes, it was sort of aligned with the Ministry of Health [initially]. But it was already behind [when it started].” (ET RS 2)

Few scientific articles were published until the end of the funding period. Despite this, CEBHA+ researchers were able to produce a range of relevant outputs [31].

### Consideration of research evidence in decision-making

Decision-makers self-reported conceptual, instrumental and tactical evidence “use” [55] in the survey. Research evidence was “considered” by decision-makers when they consulted CEBHA+ researchers: Ugandan CEBHA+ researchers were involved in the development of the Road Safety Action Plan (2021/2022–2025/2026) and were invited to present research in a parliamentary meeting on road safety, prior to the amendment of the Traffic and Road Safety Bill (30 March 2023). South African CEBHA+ researchers and their “champion” were involved in developing the National Strategic Plan for the Prevention and Control of NCDs (2022–2027).

“A key ally throughout the CEBHA + project, [decision-maker] applauded the work of CEBHA+ and linked its relevance to the National Strategic Plan for NCDs. She stressed the importance of research collaboration and the need for a coordinated platform.” (SA policy dialogue minutes)

One study by a Rwandan CEBHA+ researcher was cited in the National Strategy and Costed Action Plan for the Prevention and Control of NCDs in Rwanda (2020–2025) [56].

### Revised programme theory

**Fig. 4:**
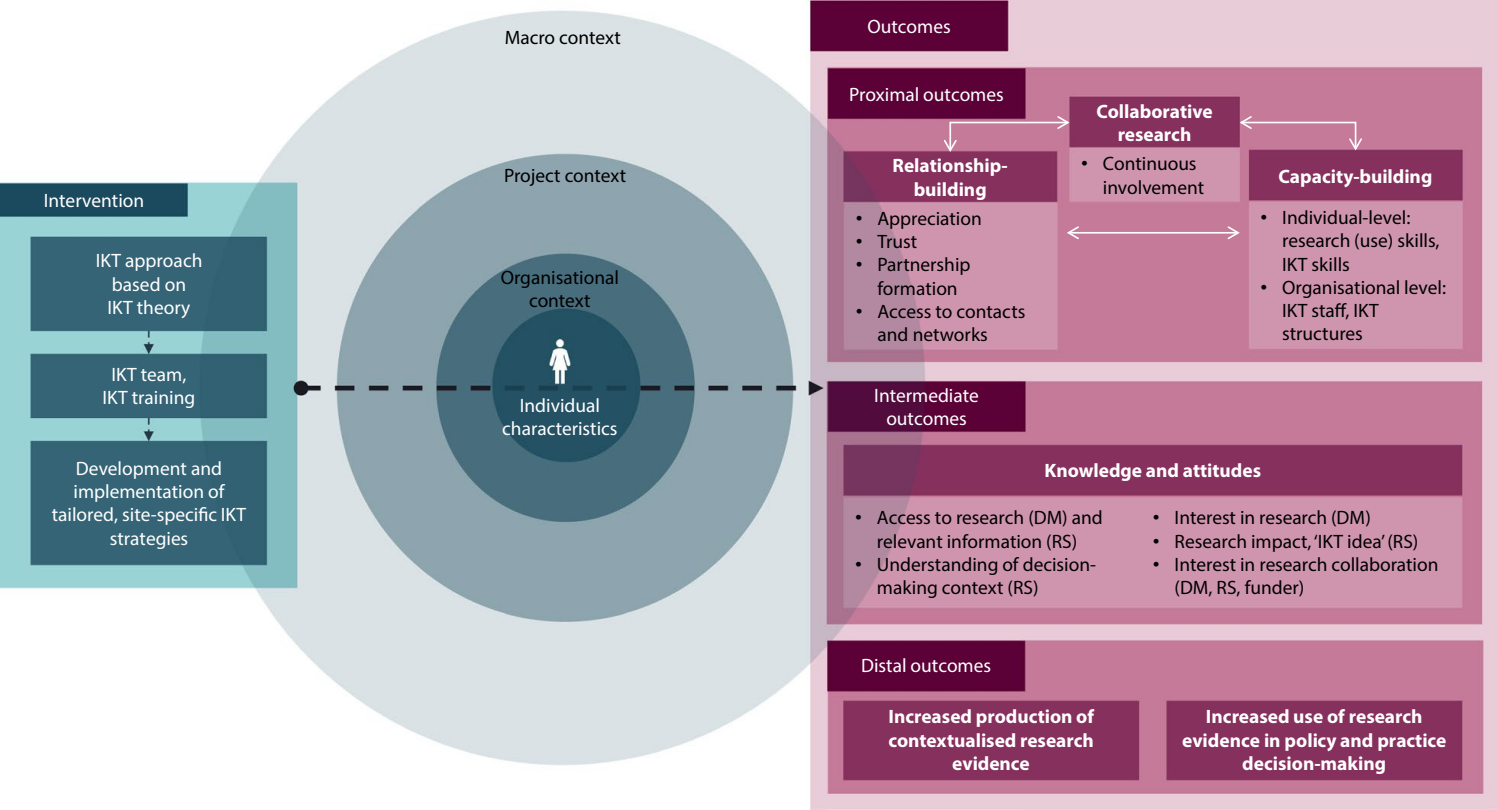
Revised programme theory of the CEBHA + IKT approach. * DM*, Decision-makers;* RS*, Researchers; macro context:* KT* Landscape, research governance, research funding, COVID-19; organizational context: KT training and structures, staff continuity, senior staff support, decision-making agenda and priorities, available resources, time, absorptive capacity, organization’s reputation, pre-existing relationships; project-specific context: IKT deliverables, resources, project priorities and timelines, complexity of the project; individual characteristics: past experience undertaking IKT/KT or collaborative research, IKT/EIDM attitudes, personal interest and motivation

The adapted programme theory includes revised subcategories. Intermediate and distal outcomes were supplemented by the researcher dimension (Fig. 4).

## Discussion

### Summary

This study examined outcomes linked to an IKT approach implemented in five African countries, drawing on a predefined programme theory. Through relationship building, capacity building and collaborative research (proximal outcomes), researchers and decision-makers built appreciation and partnerships, whilst expanding skills in conducting research, evidence use and IKT. Participants gained access to contacts and networks, but trust between partners varied. Only limited capacity was built at organizational and system levels, mostly with regards to establishing new courses and training staff in IKT. Intermediate outcomes were changes in attitudes and knowledge, particularly as researchers learned about the decision-making context. The IKT approach was linked with the production of research that was perceived as highly applicable and addressing local priorities, but we found only some evidence of consideration of evidence among decision-makers due to study limitations. In revising our initial programme theory, we were able to consolidate our findings into low-level theory.

### Contextualizing our findings in the literature

IKT emphasizes continuous relationships with knowledge users, in particular decision-makers, to foster EIDM in a mutually beneficial manner [6]. IKT activities and outcomes have been examined in a meta-synthesis of IKT case studies [57]. Whilst there remains a dearth of rigorous outcome evaluations of IKT, approaches with similar characteristics exist within the wider literature on “health research partnerships” [20], coproduction [58] and on the “science of using science” [59]. Comparison with this literature is, however, difficult due to challenges with evaluating knowledge exchange interventions in general and our approach in particular (Textbox 1).

Textbox 1: Evaluation of knowledge exchange interventions Evaluation challenges with regards to KT interventions arise from a lack of funding and other practical issues (12), its resource intensity [22], and from the complexity and context dependency of such interventions and inherent challenges to making causal claims [60]. We were faced with what has been described elsewhere as “sample-size challenge of finding enough contexts and infrastructures supporting a common approach that can be evaluated using standardized methods” [21]. Among published evaluations, there exists a positivity bias as “rich descriptions of failures” are lacking [57].

Difficulties in attributing outcomes to the IKT approach are further amplified as the approach is in itself not “one” intervention but “entails a multipronged ensemble of tailored interventions” [28]. The envisioned core components comprised continuous involvement, including through the engagement of decision-makers in priority setting for the project [51], IKT training, systematic development and implementation of “IKT strategies”, regular meetings of the IKT team and IKT-related deliverables [28]. CEBHA+ researchers were the target group for some interventions (for example IKT training) and were implementers of other interventions (implementation of IKT strategies, for example involving decision-makers in a policy dialogue). Overall, such multicomponent, multimechanism approaches are common in interventions intended to increase evidence use among decision-makers [59].

### Proximal outcomes

The CEBHA+ IKT approach was primarily aimed at individual-level behaviour change. “Relationship-building”, “capacity building” and “collaborative research” had been conceptualized to constitute the most proximal outcomes of the approach, doubling, by virtue of being activities, as mechanisms of change on the pathway to downstream outcomes [36].

CEBHA+ researchers participated in training and meetings on IKT and IKT-related skills featured prominently in capacity building. This was echoed in the meta-synthesis of IKT case studies, with “capacity for collaboration” identified as a key outcome [57]. The IKT skills we identified map onto the competencies for IKT defined in a Delphi study [60]. Interestingly, CEBHA+ researchers reported some mixed effects with respect to research skills gained, which warrants further investigation. An evidence-based public health course was implemented in CEBHA+ but since it was offered only once per country, few decision-makers participated in this formal capacity building opportunity. Given that the aim of CEBHA+ was to increase decision-makers’ evidence use, this appears to be a gap in the IKT approach. Whilst CEBHA+ activities were focused on individual-level capacity building, some strengthening of organizational and systems capacity occurred, which has been described as a key need in the region [23, 61].

Trust between partners varied and was highly context dependent, with longer relationships enabling more trusting relationships. Trust, along with “talk and time”, has been long established as key to relationship building [62]. Lately, it has been recommended, however, to view trust as a more dynamic concept in partnered research as opportunities for building trust may be different for different partners [63], as also observed in our data. Nevertheless, trust and honest communication, for example about needs, constraints and organizational realities, without concerns about judgment or backlash, are essential for building successful partnerships [64].

Decision-makers expressed great appreciation for being involved early in the research. This is more reflective of “mutually beneficial” [6], “meaningful” [20] and egalitarian partnerships [10] as opposed to one-off collaboration or work on predefined research questions, which is more likely to constitute asymmetrical or tokenistic partnerships [10]. Ambiguous use of the term “partnership” and variable levels of partnership strength pointed to all three partnership types being represented in our study [10]. We did not examine the level of individual partnerships as a unit of analysis, which we recommend for future, well-resourced evaluations. Importantly, this will help delineate partnerships with diverse groups of partners more – for example, the Ugandan team felt great tension in IKT activities involving groups with great power differentials, that is longstanding, often academic partners and others, such as the group of *boda boda* motorcycle drivers with little formal education.

CEBHA+ researchers interacted with decision-makers in particular in early and late phases in the collaborative research process, as has been documented elsewhere [1]. Whilst this may appear contradictory to the IKT tenet of “continuous” relationships, it may also reflect IKT skills, that is only involving decision-makers with a clear goal. Elsewhere, stakeholders indicated that they preferred a “moderate level of participation in research rather than full team membership”. [65]. At the same time, this may result in stakeholder roles transforming into mere research participation as opposed to consultative or coproduction roles [66].

### Intermediate outcomes

Researchers learning about the decision-making context emerged as a key outcome of the IKT approach, which had not been conceptualized in the initial programme theory. In public health, researchers have limited knowledge of policy-making [67] and therefore benefit greatly as they gain a new perspective and increase their knowledge of the policy or practice environment [1, 68] and “develop and pursue research questions that have real-world applicability, and, through ongoing conversations with decision-makers, interpret results with a deeper understanding of contextual circumstances which, in turn, enhances the usefulness of the research findings” [1]. Researchers and decision-makers generally had rather positive attitudes of collaborating, but some individuals became more convinced during the project. Such attitudinal change among decision-makers has been associated with higher frequency of research use [69, 70].

### Unintended and unanticipated effects

We found it difficult to investigate unintended, particularly adverse, effects, as interview participants appeared uncomfortable to reflect on these. This reluctance may reflect social desirability bias and added to the positivity bias common in this kind of research [57, 71], leading to us identifying predominantly positive outcomes. Challenging outcomes were identified in only 32% of reviews on health research partnerships [20], with emotional labour identified as the key negative outcome [72]. Given the substantial resources required for IKT and the various potential issues in collaborative research or coproduction [73], future evaluations should explore appropriate methods to examine this further.

### Distal outcomes

We did identify some instances of production of relevant, contextualized evidence. Such research has been reported as an important prerequisite for EIDM as it is perceived as valuable and trustworthy by local decision-makers [22]. We were further able to identify some consideration of evidence in decision-making but “evidence use” proved challenging to assess, which is a common challenge when evaluating the effect of interventions intended to facilitate EIDM on evidence use, policy and practice [74].

Despite the ambition of IKT to foster mutually beneficial relationships, the needs of evidence users – a key consideration for supporting evidence use – were only partly considered (i.e. during the priority setting for the project, when tailoring IKT activities or when undertaking stakeholder-prioritized COVID-19 research). Many IKT efforts were predominated by researchers’ needs – which is likely due to the nature of the IKT approach, as an initiative driven by researchers. This approach is further constrained by the way research is commissioned, funded and organized. In particular, short-term funding and incentives for researchers to prioritize their research over KT require better institutions, structures and longer-term funding to address evidence user needs. Systematic review facilities that focus on informing policy and practice, policy labs and other evidence-advisory institutions have been established to address this challenge [75–77].

However, efforts to strengthen evidence use in policy-making are fraught with underlying, rationalist assumptions about policy-making and evidence use [77]. In a “highly complex evidence-policy ecosystem” [77], genuinely new approaches might involve using existing local data and analysing them for policy-makers as opposed to researchers continuously creating new data, interventions and evaluations [77].

### Advancing low-level theory for IKT

Our revised programme theory can be considered a low-level theory, grounded in realist evaluation theory [36]. It links with other relevant theories on evidence use [59] and IKT [57]. The revised theory incorporates a broader consideration of the role of researchers in EIDM. This is similar to the CollaboraKTion framework but the latter is organized temporally along the research process [78, 79]. Whilst our programme theory can inform future evaluations by making assumptions about IKT explicit [35], it is by nature reductionist and may warrant stronger consideration of “systems thinking”.

### Strengths and limitations

Our study draws on a published protocol and provides unique insights into outcomes of a coordinated IKT approach implemented by teams in five African countries. We were able to enrol most CEBHA+ researchers and drew on a large number of documents. Due to the small number of decision-makers participating, we were not able to disaggregate survey results by country. The under-representation of decision-makers may present a finding in itself: In some cases, researchers advised against the enrolment of their decision-making partners, for example when the latter were overworked with COVID-19-related responsibilities or when researchers deemed that partnerships were not sufficiently established to link partners with evaluation researchers.

The comparative case study design does not allow for causal inference. However, in the absence of an opportunity to employ a more rigorous study design to examine effects, the study design still allows for teasing out whether the approach contributed to some change in the direction of the hypothesized outcomes.

We used a nonvalidated survey tool, which incorporated validated constructs from existing tools. This led to some conceptual difficulties at the data analysis stage and we recommend the use of other tools for future IKT research [18]. Some relevant concepts, such as organizational absorptive capacity, were underexamined. Our data analysis included recommended double-coding by one researcher [43] but restricts the perspective, compared with double-coding by multiple researchers. Further limitations arise from the involvement of evaluators in IKT implementation, as we discuss under reflexivity, and from the risk of social desirability bias given the proximity of evaluators and participants.

## Conclusions

Our evaluation of an IKT approach implemented in five African countries presents theory-informed insights into proximal, intermediate and distal IKT outcomes, based on participants’ views and documented IKT activities. Given the multicomponent nature and context dependency of KT interventions as well as the diversity of relevant decision-makers in public health and healthcare there is not one way for implementers to “do IKT”. IKT evaluation literature can certainly inform future IKT efforts. However, implementers must (i) take genuine consideration of decision-makers’ needs and preferences, and (ii) monitor their IKT activities to understand “what works” in their context. This should include a cost–benefit-assessment of KT activities, in particular in resource-constrained settings [68, 80]. Research on unintended effects of IKT, including those arising from power differentials [81], is warranted. Importantly, beyond project-based, often haphazard IKT, evidence-informed decision-making requires strengthening of structures and systems.

## Supplementary Information

Supplementary material 1. ASSESS tool.

Supplementary material 2. Collaborative research table.

Supplementary material 3. Coding frame.

## Data Availability

The datasets generated and analysed during the current study are not publicly available due to concerns about anonymity of study participants given the small sample size. Survey data can be made available from the corresponding author on reasonable request.

## Abbreviations

IKT: Integrated knowledge translation
KT: Knowledge translation
CEBHA+: Collaboration for Evidence-Based Healthcare and Public Health in Africa
LMU: Ludwig-Maximilians Universitaet Muenchen
EIDM: Evidence-informed decision-making
COVID-19: Coronavirus disease 2019
FGD: Focus group discussion
NCD: Noncommunicable diseases
ET: Ethiopia
MW: Malawi
RW: Rwanda
SA: South Africa
UG: Uganda
RS: Researcher
SH: Stakeholder
DM: Decision-maker
AHRI: Armauer–Hansen research institute
MOH: Ministry of Health

## Footnotes

1 IKT is defined as an “ongoing relationship between researchers and decision-makers” (1) and is distinct from broader “knowledge user” or “stakeholder” engagement. However, some IKT strategies included stakeholders beyond decision-makers [for example academic or nongovernmental organization (NGO) staff], which is why we use both terms interchangeably.

2 Country omitted to maintain anonymity.
