# Supplementary material for: Outcomes of an integrated knowledge translation approach in five African countries: a mixed-methods comparative case study
Source: Health Res Policy Syst. 2024 Dec 10;22:162. doi: 10.1186/s12961-024-01256-x (PMC11629502; doi:10.1186/s12961-024-01256-x)
Supplement: Supplementary file 1 — Supplementary material 1. ASSESS tool. [file 12961_2024_1256_MOESM1_ESM.docx]

| **Research phase** |  | | **Ethiopia** | | **Malawi** | | **Rwanda** | | **South Africa** | | **Uganda** | |
| --- | --- | --- | --- | --- | --- | --- | --- | --- | --- | --- | --- | --- |
|  | Total n quotations per country | | 245 |  | 518 |  | 384 |  | 700 |  | 658 |  |
|  | Total n survey responses (all waves) ^a,b^ | |  | 9 |  | 16 |  | 16 |  | 15 |  | 13 |
|  |  | | Qual: code frequency | Survey:  n agree (%) | Qual: code frequency | Survey:  n agree (%) | Qual: code frequency | Survey:  n agree (%) | Qual: code frequency | Survey:  n agree (%) | Qual: code frequency | Survey:  n agree (%) |
| **Early** | **Choosing research topic (broad) ^a^** | No | 2 |  | 7 |  | 0 |  | 0 |  | 0 |  |
|  |  | Yes | 3 | 1 (50)^a^ | 8 | 2 (67)^a^ | 3 | 1 (25)^a^ | 10 | NA | 9 | 0 |
|  | **Defining research question or methods** | No | 1 |  | 4 |  | 0 |  | 2 |  | 0 |  |
|  |  | Yes | 2 | 6 (67) | 5 | 11 (69) | 9 | 9 (56) | 9 | 11 (73) | 9 | 6 (46) |
| **Intermediate** | **Joint data collection** | No | 0 |  | 0 |  | 0 |  | 0 |  | 0 |  |
|  |  | Yes | 1 | 4 (44) | 1 | 5 (31) | 9 | 10 (63) | 2 | 5 (33) | 1 | 8 (62) |
|  | **Facilitated data collection** | No | 0 |  | 0 |  | 0 |  | 0 |  | 0 |  |
|  |  | Yes | 4 |  | 0 |  | 16 |  | 0 |  | 13 |  |
|  | **Data analysis** | No | 0 |  | 0 |  | 0 |  | 0 |  | 1 |  |
|  |  | Yes | 0 | 3 (33) | 0 | 3 (19) | 2 | 7 (44) | 0 | 4 (27) | 1 | 2 (15) |
| **Late** | **Data interpretation** | No | 0 |  | 1 |  | 0 |  | 0 |  | 0 |  |
|  |  | Yes | 0 | 3 (33) | 1 | 8 (50) | 3 | 13 (81) | 1 | 12 (80) | 6 | 9 (69) |
|  | **Publication** | No | 0 |  | 0 |  | 0 |  | 0 |  | 0 |  |
|  |  | Yes | 0 |  | 0 |  | 9 |  | 0 |  | 0 |  |
|  | **Dissemination ^a^** | No | 0 |  | 0 |  | 0 |  | 1 |  | 0 |  |
|  |  | Yes | 9 | 0 | 28 | 1 (33)^a^ | 2 | 3 (75)^a^ | 4 | NA | 21 | 2 (100)^a^ |
|  | **Act on results** | No | 0 |  | 0 |  | 0 |  | 0 |  | 6 |  |
|  |  | Yes | 2 |  | 0 |  | 1 |  | 0 |  | 7 |  |
| **Legend**: Frequencies of quotations (qualitative data) and agreement rates with survey items (quantitative data) indicating whether collaboration had (green shading) or had not (orange shading) occurred at certain stages of the research process. Darker shading higher frequencies and agreement rates. Participants from the same country may make contrasting statements.  ^a^ the items ‘choosing research topic’ and ‘dissemination’ were only included in the DM 2022 surveys (lower number of responses for these items)  ^b^ aggregated responses form decision-makers and researchers from one country  Gr: grounded = overall number of quotations coded by one code. | | | | | | | | | | | | |
